# Examining the gender difference in the association between metabolic syndrome and the mean leukocyte telomere length

**DOI:** 10.1371/journal.pone.0180687

**Published:** 2017-07-07

**Authors:** Yuan-Yuei Cheng, Tung-Wei Kao, Yaw-Wen Chang, Chen-Jung Wu, Tao-Chun Peng, Li-Wei Wu, Hui-Fang Yang, Fang-Yih Liaw, Wei-Liang Chen

**Affiliations:** 1 Division of Family Medicine, Department of Family and Community Medicine, Tri-Service General Hospital and School of Medicine, National Defense Medical Center, Taipei, Taiwan, Republic of China; 2 Division of Geriatric Medicine, Department of Family and Community Medicine, Tri-Service General Hospital and School of Medicine, National Defense Medical Center, Taipei, Taiwan, Republic of China; 3 Graduate Institute of Medical Sciences, National Defense Medical Center, Taipei, Republic of China; 4 Division of Family Medicine, Department of Community Medicine, Taoyuan Armed Forces General Hospital, Taoyuan, Taiwan, Republic of China; Universita degli Studi di Milano, ITALY

## Abstract

The mechanism of cellular aging likely involves decreased telomere length and is associated with age-related diseases such as cardiovascular disease. Metabolic syndrome (MetS) is an important risk factor for CVD. The purpose of this study was to investigate the association between LTL and MetS. We evaluated 7370 participants in the National Health and Nutrition Examination Survey (1999–2002). The association between LTL and individual MetS components and the number of MetS components was analyzed by multivariable regression models, adjusting for gender, race/ethnicity, albumin, C-reactive protein, alanine transaminase, uric acid and medical condition. An increase in the number of MetS components was strongly associated with shorter telomere length, especially in female participants (p for trend < 0.05). In addition, triglycerides were negatively associated with LTL in female participants (p < 0.001). Waist circumstance was associated with decreased LTL (p < 0.05) in both males and females. In summary, our study indicated that an increment of MetS component is strongly associated with shorter LTL, especially in the female population.

## Introduction

Telomere are specific DNA–protein structures found at both ends of each chromosome and are essential and dynamic regulators of cellular life span and chromosome integrity[1]. The mean leukocyte telomere length is related to cellular aging[2]. LTL shortening is strongly associated with certain types of age-related diseases such as cardiovascular disease, coronary disease, diabetes mellitus and all-cause mortality[3]. Short telomeres can result in cellular senescence and apoptosis which contribute to the development of atherosclerosis and a predisposition to plaque instability[4]. In contrast, longer telomeres are associated with increased survival, especially among men and those who are active and the importance of advance of physical activity behavior is stressed[5]. Aging is characterized by shortening of telomere length and also is also associated with cardiovascular disease[6]. A Japanese study of patients with DM or impaired glucose tolerance demonstrated that excessive oxidative stress leads to telomere damage reduced telomere length[7].

Metabolic syndrome (MetS) is defined by the Modified National Cholesterol Education Program-Adult Treatment Panel III criteria as a constellation of abdominal obesity, dyslipidemia, hypertension and insulin resistance[8]. Metabolic syndrome is highly prevalent in the elderly population in the US[9]. A study by Veronica et al revealed that aging is accelerated when metabolic and cardiovascular diseases are present and that the risk of these diseases increases with age[10]. Another study, indicated that the pathogenesis of metabolic syndrome and related diseases involves excess reactive oxygen species, which damage mitochondrial components and trigger cellular lysis[11]. Such toxic reactions lead to the aging process.

The association of individual MetS components with shorter LTL had been examined in several previous studies. For glucose, high oxidative stress potentially lead to accelerated telomere shortening in type 2 diabetes mellitus development[12]. In a longitudinal study reported by Lee et al, telomere length was shorter in an obese population[13]. In a cohort study of 5,598 participants, shorter LTL was associated with multiple measures of obesity in both males and females[14]. In terms of high blood pressure, leukocyte telomeres may be shorter in hypertensive than in normotensive individuals. Rehkopf and his colleagues showed that triglyceride was a risk factors for CVD and CHD that is strongly associated with shorter LTL[15]. Shorter telomere length, a cellular marker for biological age, was associated with a higher metabolic risk profiles, which remains unfavorable even after a period of 6 years[16]. Accumulating evidences demonstrated that shortened leukocyte telomere length had a significant association with stroke, myocardial infarction, and type 2 diabetes mellitus[17–19]. However, it is unclear whether gender differences existed in the association between MetS and leukocyte telomere length. Our aim was to explore the gender differences in relationship between LTL and MetS by analyzing data obtained from the National Health and Nutrition Examination Survey (NHANES) between 1999 and 2002.

## Methods

### Ethics statement

The NHANES study protocol was based on the National Center for Health Statistics Institutional Review Board (IRB). Before data collection procedures and examinations, all eligible participants were asked to complete the consent forms agreeing to participate in the survey.

### Study design and participants

We collected data from the National Health and Nutrition Examination Survey (NHANES), which consisted of a detailed home interview and a health examination conducted by the National Center for Health Statistics (NCHS) of the Centers for Disease Control and Prevention. We analyzed these data which are available for public download (http://www.cdc.gov/nchs/nhanes/nhanes_questionnaires.htm). Comprehensive studies, including demographic data, laboratory results, questionnaire contents, and mean leukocyte telomere length, were collected from 2 NHANES datasets (1999–2000 and 2001–2002). Participants who lacked associated results of laboratory and clinical examinations were excluded, such as the components of metabolic syndrome and mean leukocyte telomere length.

### Definition of MetS

As defined by the Modified National Cholesterol Education Program–Adult Treatment Panel III criteria, MetS was diagnosed if an individual exhibited 3 or more of the following components: (1) waist circumstance ≥ 90th percentile for age and sex; (2) triglyceride ≥ 110 mg/dL; (3) HDL-C ≤ 40 mg/dL; (4) systolic blood pressure or diastolic blood pressure ≥ 90th percentile for age, sex, and height, use of BP-lowering medication or a previous diagnosis of hypertension; and (5) fasting plasma glucose ≥ 100 mg/dL, current diabetes status, or current use of insulin or hypoglycemic medication[8].

### Mean leukocyte telomere length

Blood samples were obtained from the laboratory at NHANES, National Center for Health Statistics, Centers for Disease Control and Prevention. Using standardized procedures, DNA was purified from whole blood and stored at −80°. The telomere length assay was performed in the laboratory using the quantitative PCR method to measure telomere length relative to standard reference DNA (T/S ratio)[20, 21]. Control DNA values were used to normalize between-run variability. Runs in which more than four control DNA values fell outside 2.5 standard deviations from the mean for all assay runs were excluded from further analysis (<6% of runs). The formula of conversion of T/S ratio to base pairs (bp) was (3,274 + 2,413 * (T/S)). The conversion from T/S ratio to bp was calculated based on comparison of telomeric restriction fragment (TRF) length from Southern blot analysis and T/S ratios using DNA samples from the human diploid fibroblast cell line IMR90 at different population doublings[22].

### Covariates

We analyzed laboratory data such as serum uric acid, total bilirubin, total cholesterol, triglycerides, HDL-C cholesterol, LDL cholesterol, serum glucose, C-reactive protein, waist circumstance and blood pressure. The details of the measurement of these factors are described in the NHANES documentation. All protocols were conducted with standard method according to the CDC reference, and all data was downloaded from the NHANES website. We acquired demographic variables such as age, sex, race/ethnicity, smoking history, and medical status from self-reported data. The interviewer asked the question “Do you now smoke cigarettes” to confirm the smoking status of the respondents. We obtained the medical history base on whether by they had ever been diagnosed with or told by doctors and professionals that they had coronary artery disease, heart attack, congestive heart failure, angina, cancer and stroke.

### Statistical analysis

Because of the complicated survey design used in the National Health and Nutrition Examination Survey III, conventional calculations of statistical analyses according to the assumption of a simple random sample would provid improper variance estimates and thus are not appropriate. All statistical analyses were performed in SPSS (Version 18.0 for Windows, SPSS, Inc., Chicago, IL, USA). We used multivariable regression models to assess the association between LTL and the number of MetS components or each individual MetS component, and they were used in covariate adjustment. Model 1 consisted of age, sex, and race/ethnicity adjustment. In Model 2, Model 1, albumin, C-reactive protein, alanine transaminase (ALT) and uric acid adjustment were included. Model 3 included Model 2 and heart failure, coronary artery disease (CAD), heart attack, angina, stroke, cancer and smoking status adjustment. To explore the association between an increased number of MetS components and mean leukocyte telomere length, P-values for the trend were used by regarding the number of MetS components as a continuous variable. Two-sided P-values < 0.05 were considered to indicate significant differences.

## Results

### Sample characteristics

**Fig 1:**
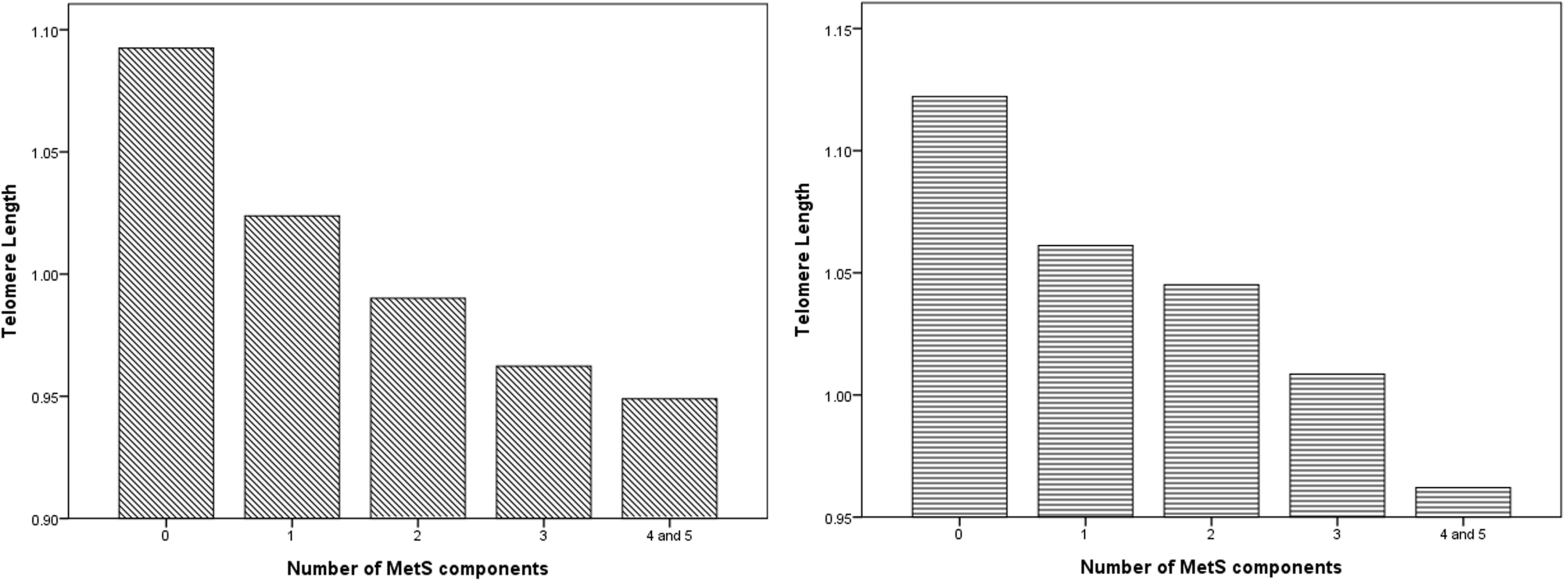
(A) distribution of mean leukocyte telomere length according to the number of MetS components in male participants. (B) distribution of mean leukocyte telomere length according to the number of MetS components in female participants.

We used a stratified sampling method to divide participants according to the presence of metabolic syndrome and gender in Table 1. In the study, the mean age of participants with metabolic syndrome was 56.57±16.25 years for males and 56.61±17.58 years for females. In the non-metabolic syndrome group, the mean age was 47.88±18.45 years for males and 44.57±18.36 years for females. Blood pressure, waist circumstance, serum triglycerides, serum glucose, serum ALT, serum uric acid, C-reactive protein and self-reported medical condition/smoking history were significantly greater in MetS participants than non-MetS participants. As expected, serum HDL-cholesterol was significantly lower in MetS participants than non-MetS participants. In the Fig 1, it existed a decreasing relationship in mean LTL with an increase in the numbers of components of MetS in the male (Fig 1A) and female (Fig 1B) participants (p for trend < 0.05).

**Table 1:** Characteristics of participants with or without metabolic syndrome.

Variables	Men (N = 3564)			Women(N = 3806)		
	Non-Metabolic syndrome	Metabolic syndrome	P-value	Non-Metabolic syndrome	Metabolic syndrome	P-value
**Continuous Variables, mean (SD)**						
Mean Telomere Length (T/S ratio)	1.033(0.31)	0.957(0.24)	<0.001	1.070(0.27)	0.990(0.24)	<0.001
Age (years)	47.88(18.45)	56.57(16.25)	<0.001	44.57(18.36)	56.61(17.58)	<0.001
SBP(mmHg)	125.35(17.61)	137.07(20.99)	<0.001	120.66(21.87)	139.11(24.69)	<0.001
DBP(mmHg)	72.39(13.68)	76.62(16.02)	<0.001	68.38(12.61)	72.29(15.18)	<0.001
Waist circumference (cm)	95.31(12.72)	110.40(12.28)	<0.001	90.56(14.10)	104.55(13.34)	<0.001
Serum triglycerides (mg/dL)	122.01(86.94)	250.40(226.81)	<0.001	109.57(58.36)	211.98(129.42)	<0.001
HDL-C (mg/dL)	49.66(13.46)	37.66(8.70)	<0.001	60.81(15.27)	46.41(12.70)	<0.001
Serum glucose (mg/dL)	93.42(24.67)	117.48(52.56)	<0.001	86.67(18.23)	112.64(50.31)	<0.001
Serum albumin (g/dL)	4.46(0.32)	4.34(0.32)	<0.001	4.21(0.37)	4.17(0.32)	<0.001
Serum ALT (U/L)	29.30(32.27)	33.89(23.55)	<0.001	20.45(19.43)	24.92(58.29)	<0.001
Serum uric acid (mg/dL)	5.93(1.25)	6.42(1.47)	<0.001	4.40(1.20)	5.29(1.46)	<0.001
C-reactive protein (mg/dL)	0.35(0.77)	0.49(0.98)	<0.001	0.48(0.95)	0.72(0.86)	<0.001
**Categorical variables, n (%)**						
Race-ethnicity, %			<0.001			0.005
Mexican American	609(23.5)	253(26.1)		603(22.5)	318(28.1)	
Other Hispanic	125(4.8)	49(5.1)		157(5.9)	58(5.1)	
Non-Hispanic White	1316(50.7)	529(54.6)		1372(51.3)	551(48.8)	
Non-Hispanic Black	467(18.0)	118(12.2)		459(17.2)	166(14.7)	
Other	78(3.0)	20(2.1)		85(3.2)	37(3.3)	
Education Level						
More than high school	1739 (67.0)	584 (60.3)	<0.001	1913 (71.5)	681 (60.3)	<0.001
Less than high school	855 (33.0)	385 (39.7)		762 (28.5)	449 (39.7)	
Annual family income (dollars)			0.084			<0.001
≥$20,000	1798 (71.6)	638 (68.5)		1791 (69.2)	630 (58.3)	
<$20,000	714 (28.4)	293 (31.5)		798 (30.8)	450 (41.7)	
Congestive heart failure	63(2.4)	49(5.1)	<0.001	45(1.7)	47(4.2)	<0.001
Coronary heart disease	113(4.4)	88(9.1)	<0.001	51(1.9)	53(4.7)	<0.001
Angina	80(3.1)	67(6.9)	<0.001	53(2.0)	61(5.4)	<0.001
Heart attack	126(4.9)	81(8.4)	<0.001	42(1.6)	54(4.8)	<0.001
Stroke	50(1.9)	59(6.1)	<0.001	47(1.8)	51(4.5)	<0.001
Cancer	218(8.4)	82(8.5)	<0.001	191(7.1)	116(10.3)	<0.001
Smoke	1488(57.4)	627(64.7)	<0.001	1004(37.5)	462(40.9)	<0.001

SD, standard deviation; SBP, systolic blood pressure; DBP, diastolic blood pressure; HDL-C, high-density lipoprotein cholesterol; ALT, alanine aminotransferase

### Metabolic syndrome and mean leukocyte telomere length

**Fig 2:**
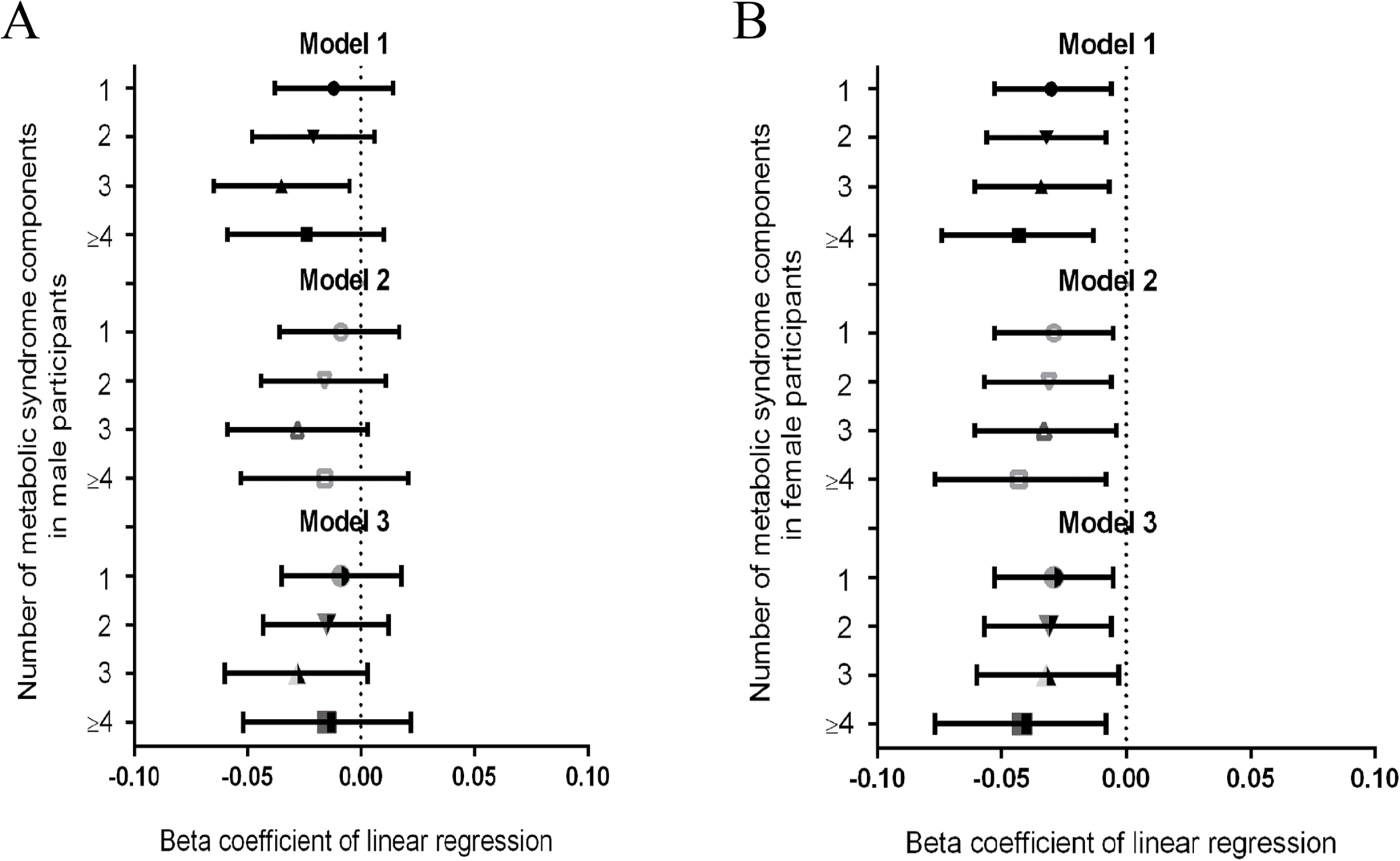
(A) Relationship between the number of metabolic syndrome components and mean telomere length in male participants. (B) Relationship between the number of metabolic syndrome components and mean telomere length in female participants.

After additional adjustment (Fig 2A), the β coefficient of the mean LTL considering 1, 2, 3 and >4 MetS components was -0.029, -0.034, -0.032, -0.044 in Model 3 for females (p for trend = 0.012). However, as shown in the regression model, mean LTL was not associated with significantly increased MetS components (Fig 2B). There was a strong linear decrease in the mean LTL when the number of metabolic syndrome components increased among females. Table 2 and Table 3 present the regression coefficients for the association of the components of MetS with mean LTL in males and females.

**Table 2:** Regression coefficients of components of metabolic syndrome for mean Telomere length in male participants.

Variables	Model 1		Model 2		Model 3	
	β (95% CI)	P-value	β (95% CI)	P-value	β (95% CI)	P-value
Components of metabolic syndrome						
Waist circumference	-0.024(-0.043, -0.006)	0.011	-0.022(-0.041, -0.003)	0.024	-0.022(-0.041, -0.003)	0.025
Blood pressure	0.003(-0.017, 0.023)	0.755	0.004(-0.016, 0.023)	0.723	0.003(-0.017, 0.023)	0.761
Low HDL-C	-0.008(-0.028, 0.011)	0.389	-0.006(-0.026, 0.013)	0.536	-0.006(-0.025, 0.014)	0.561
High triglycerides	-0.007(-0.026, 0.012)	0.483	-0.003(-0.022, 0.016)	0.731	-0.003(-0.022, 0.016)	0.757
High glucose	-0.010(-0.032, 0.011)	0.341	-0.017(-0.029, -0.014)	0.492	-0.008(-0.029, -0.014)	0.468

Model 1 = age, race-ethnicity, education level, annual family income

Model 2 = Model 1+ (serum albumin, C-reactive protein, serum ALT, serum uric acid).

Model 3 = Model 2+ (heart failure, CAD, angina, heart attack, stroke, cancer and smoke)

**Table 3:** Regression coefficients of components of metabolic syndrome for mean Telomere length in female participants.

Variables	Model 1		Model 2		Model 3	
	β (95% CI)	P-value	β (95% CI)	P-value	β (95% CI)	P-value
Components of metabolic syndrome						
Waist circumference	-0.025(-0.042, -0.009)	0.002	-0.025(-0.042, -0.007)	0.007	-0.025(-0.042, -0.007)	0.007
Blood pressure	0.012(-0.007, 0.032)	0.222	0.013(-0.007, 0.033)	0.206	0.013(-0.007, 0.033)	0.206
Low HDL-C	-0.009(-0.025, 0.007)	0.289	-0.007(-0.024, 0.009)	0.392	-0.007(-0.024, 0.009)	0.400
High triglycerides	-0.032(-0.049, -0.015)	<0.001	-0.031(-0.049, -0.014)	<0.001	-0.031(-0.048, -0.014)	<0.001
High glucose	-0.010(-0.031, 0.011)	0.357	-0.007(-0.028, 0.014)	0.513	-0.007(-0.029, 0.014)	0.505

Model 1 = age, race-ethnicity, education level, annual family income

Model 2 = Model 1+ (serum albumin, C-reactive protein, serum ALT, serum uric acid).

Model 3 = Model 2+ (heart failure, CAD, heart attack, angina, stroke, cancer, smoke)

As shown in Table 3, in female participants, high triglyceride was highly significantly associated with a decrease in mean LTL in fully adjusted models (p < 0.001). In both the male and female groups (Table 2 and Table 3), waist circumstance was significantly associated with a decrease of mean LTL (p < 0.05).

## Discussion

In our study, shorter LTL was favorably associated with metabolic health risk, particularly the criteria for MetS. Notably, an increment in the number of MetS components in female participants had a strong linear association with shorter LTL. This study indicates that a better understanding of the clustering of metabolic syndrome will facilitate the elucidation of the link between LTL and metabolic risk factors. This is the first large-scale study to demonstrate gender differences in the relationship between MetS components and shorter mean telomere length.

A prospective cohort study, by Révész et al, explored the longitudinal associations between MetS dysregulations and telomere length over a 6-year period which revealed that higher baseline waist circumference, blood glucose and lower HDL cholesterol were significantly associated with shorter telomere length[3]. A previous study reported that an one unit difference in the following biomarkers were associated with kilobase pair differences in LTL, such as BMI, waist circumference, percentage of body fat, HDL, triglycerides, pulse rate, systolic blood pressure and diastolic blood pressure[15]. However, these studies only focused on the relationship between individual MetS components and telomere length. No further analysis of the number of MetS components influencing shorter telomere length was performed. Our regression analysis supports a significant inverse dose-response relationship between shorter telomere length and the number of MetS components, especially in females.

In the present study, waist circumstance in both men and women was significantly associated with shorter LTL. A cross-sectional analysis of 21,004 participants of all ages revealed a 0.2% decrease in telomere length for every kg/m^2^ increase in BMI, whereas a unit increase in waist circumference and percent body fat contributed to a decreases in LTL of 0.09% and 0.01% respectively[23]. A substantial body of research indicates that abdominal adiposity is positively related to high levels of biomarkers of oxidative stress and low systemic inflammation[24–26]. Adipose tissue may stimulate inflammation by releasing proinflammatory mediators and increasing levels of adipokines, especially leptin[27]. Increased plasma leptin level in obese and lean participants may promote the production of IL-6, CRP, and other proinflammatory cytokines, which regulate insulin sensitivity and exert inflammatory activities[22]. It is tempting to speculate that adipose tissue may be an important source of systemic chronic inflammation, leading to shorter telomere length[28].

A cross-sectional study of US adults, by Rehkopf et al, demonstrated that triglyceride is a risk factor for cardiovascular disease and is strongly associated with shorter LTL[15]. A 10-year prospective study from 1995 to 2005 revealed that baseline telomere length in bariatric patients was inversely associated with plasma triglycerides concentration[29]. The triglyceride level is one of several lipid parameters that can aid the prediction of coronary heart disease (CHD) risk, and elevated plasma TG levels are strongly associated with an increased risk of CHD[30]. The previous studies are consistent with our findings of a significant association between high triglyceride level and shorter telomere length in females but not males. Elevated serum triglyceride levels have been shown to play an important role in the pathogenesis of insulin resistance, and the insulin resistance- hyperinsulinemic- metabolic complex elevates metabolic health and cardiovascular risks[31]. The current consensus is that inflammation and oxidative stress are the unifying factors that explain the association of a relatively short LTL with atherosclerosis and with insulin resistance[32, 33]. In mice, short telomeres result in metabolic dysfunction through mitochondrial dysfunction[34], whereas mice with disruption of Rap1, a telomere-binding protein, exhibit accumulation of abdominal fat and insulin resistance[35, 36]. A previous study, by Zhou et al, reported the interaction between short telomeres and T2DM risk appears to involve mitochondrial dysfunction as an intermediate process[37]. The hypertriglyceridemia mechanism underlying the association of insulin resistance with telomere length is poorly understood.

In the present study, an increasing number of MetS components was strongly associated with shorter telomere length, especially in females. The mean age of the MetS female group was approximately 56 years. In a prospective study of a population of 486 white elderly females, women with the longest leukocyte telomere length underwent menopause three years later than those with the shortest leukocyte telomere length[38]. In addition, a recent Korean study demonstrated that decreased estrogen levels after menopause are related to shorter telomere length compared to the premenopausal state[39]. Kyo and his colleagues examined the existence of hormone-dependent mechanisms by which estrogen may affect telomerase activity[40]. Telomerase is a reverse transcriptase enzyme that can prevent telomere shorting[1]. Another study demonstrated that LTL were significantly higher in postmenopausal female population who had received long-term hormone therapy with estrogen than in female who had never taken estrogen after menopause[41]. The decrease in estrogen levels in postmenopausal females compared to premenopause may prevent activation of telomerase to catalyze the synthesis and extension of telomeres[39]. For the relationship between LTL and testosterone, one previous study performed by Wang et al, reported LTL were positively associated with polycystic ovary syndrome(PCOS) and indicated higher testosterone expression could promote telomere lengthening[42]. In a prospective clinical study, patients with short telomeres had an increase in telomere length in response to a pharmacologic intervention with male hormone[43]. Yeap et al reported that serum dihydrotestosterone and estrodiol correlated with LTL independently of chronological age, though aromatase gene polymorphisms are associated with both lower serum estrodiol and shorter LTL in community-dwelling men[44]. Sex hormones may be useful in the treatment of telomere attrition, such as post-chemotherapy[45] and hematopoietic stem cell transplantation[46].

There are some potential limitations in the present study that warrants consideration. First, causal inference is not suitable because we cannot explain whether the MetS components affect the shortening of LTL; a longitudinal study would be more convincing than a cross-sectional study. Second, the technique for measuring telomere length can lead to bias in measurements because of the high variability of qPCR analysis. Consequently, potential experimental errors should be carefully controlled. Third, a potential for recall bias exists because of self-reported method of past medical histories. Fourth, information on pharmacological treatment was lacking in our study, such as post-menopausal or hormone-replacement therapy. The plausible relevance of estrogen metabolism on LT and its association with metabolic derangement were not established. In the future, it might be the emphasis to assess whether decreased metabolic components postpone the reduction of telomere length. Finally, limited ethnicity diversity in the participants were enrolled from 1999 to 2002, which may not reflect current racial distribution among the United States' population. However, according to a previous study of the prevalence of MetS in the US[47], MetS remains widespread in older and non-Hispanic whites, consistent with our sample.

## Conclusion

In summary, the present study demonstrates a strong linear association between an increment in the number of MetS components and shorter telomere length, especially in female. Additionally, individual MetS component such as waist circumstance and high triglyceride were inversely associated with shorter telomere length. Our findings provide epidemiological information for further studies to determine the molecular mechanisms of underlying telomere length and the development of interventions for preventing disease progression.
